# Scientific rationale and practical realities of the 140–160 bpm national standard in Chinese school physical education for primary and middle school students

**DOI:** 10.3389/fpubh.2025.1720764

**Published:** 2025-11-19

**Authors:** Ziquan Zhang, Yunping Xia, Bo Liu, Yunxiang Fan

**Affiliations:** College of Physical Education, Hunan Normal University, Changsha, China

**Keywords:** child and adolescent health, exercise intensity, moderate-to-vigorous physical activity, heart rate, public health policy

## Abstract

**Objectives:**

To examine the scientific rationale, implementation realities, and policy significance of China’s new Compulsory Education Physical Education and Health Curriculum Standard specifying an average in-class heart rate (HR) of 140–160 bpm.

**Method:**

Narrative policy and literature analysis using official Ministry of Education documents, international guidelines, and empirical studies on youth HR and moderate-to-vigorous physical activity (MVPA).

**Findings:**

The 140–160 bpm range equals roughly 65%–80% of age-predicted HRmax, aligning with international MVPA definitions. Early school-based evidence shows uneven attainment due to equipment, training, and interpretive gaps.

**Conclusion:**

The standard translates exercise science into educational governance but requires strengthened teacher preparation, digital infrastructure, and equitable funding to achieve sustainable, health-oriented reform.

## 1 Introduction

The Compulsory Education Physical Education and Health Curriculum Standards (2022 Edition) (hereafter, the 2022 Standard) mark a decisive milestone in China’s educational reform, embodying the national commitment to the principle of “Health First” (1). Developed under the leadership of the Ministry of Education, the 2022 Standard integrates physical, psychological, and moral development in accordance with the principle of moral cultivation. It encourages active participation through the pedagogical triad of “learning, practicing, and competing” regularly, aiming to improve children’s physical literacy, resilience, and lifelong engagement in physical activity. Within this framework, physical education (PE) is positioned as both an instrument for fitness enhancement and a foundation for sustainable health literacy and personal growth. Building on this foundation, the formulation of the 2022 Standard also responded to long-standing national debates about how to make PE more effective in improving real health outcomes.

A defining innovation of the 2022 Standard is its explicit quantification of exercise intensity. It recommends that each PE class achieve moderate-to-vigorous intensity, with students’ average HR maintained between 140 and 160 beats per minute (bpm). This is the first national effort to integrate a physiological target directly into educational evaluation. The introduction of this quantifiable physiological benchmark was not arbitrary but the culmination of two decades of national concern about insufficient physical activity intensity among students. Repeated government surveys had revealed that many PE classes remained “non-sweating” (2) with students seldom reaching moderate exercise levels. These findings, together with the “Health First” education principle and the observed decline in youth fitness indices since the early 2000s (3), prompted policymakers to seek a scientific mechanism to ensure adequate exertion. The new HR target was thus conceived as a way to transform curriculum oversight from subjective observation to measurable engagement. The expectation is that the standard will help teachers better manage lesson intensity, promote equitable physical-fitness improvement, and foster lifelong health literacy. By introducing a fixed heart rate (HR) target, the policy aspires to promote scientific load control, ensure sufficient exercise engagement, and address long-standing concerns about “low-activity” PE classes (2). Although the standard applies a uniform range, calculations based on established age-related maximal HR (HRmax) equations suggest that this target roughly corresponds to 65–85 percent of HRmax for most primary and middle-school students—well within the internationally recognized moderate-to-vigorous zone. From this perspective, the 2022 Standard deserves credit as a bold, evidence-informed attempt to align school-based physical activity with developmental physiology.

At the same time, the introduction of a national HR benchmark reveals the inherent tension between scientific precision (4, 5) and policy pragmatism. Children’s cardiovascular responses vary with age, sex, maturation, and contextual factors, making individualized calibration impractical within large-scale public systems. The 140–160 bpm range should therefore be viewed less as an exact prescription than as a normative guide—encouraging teachers and schools to design lessons that elicit meaningful physical exertion. This pragmatic balance between direction and precision reflects both the ambition and the constraints of health-oriented educational policy.

The emergence of the 2022 Standard thus represents a critical step in translating exercise science into educational governance. Its HR target may not capture every individual difference, yet it introduces a measurable physiological dimension into a field historically assessed by behavioral and pedagogical indicators. In this respect, the 2022 Standard establishes an important conceptual bridge between health science and pedagogy—one that merits both recognition and constructive refinement.

To critically evaluate this innovation, we conducted a narrative evidence synthesis drawing on official documents from the Chinese Ministry of Education, World Health Organization, as well as peer-reviewed literature indexed in PubMed and China National Knowledge Infrastructure (2000–2025). Search terms included “school physical education,” “heart rate,” “exercise intensity,” and “children or adolescents.” Sources were selected for their relevance to physiological load, policy implementation, or comparative international standards. The purpose of this policy brief is therefore to examine the physiological rationale, practical implications, and implementation challenges of the 140–160 bpm target, situating China’s policy within the broader context of international pediatric exercise science and education.

## 2 Scientific foundations of the 140–160 bpm standard

The integration of a quantifiable exercise-intensity benchmark in the 2022 Standard reflects a growing global recognition that school-based PE should move beyond descriptive participation toward physiologically informed practice. For children and adolescents, moderate-to-vigorous physical activity (MVPA) represents the critical intensity threshold for cardiovascular and developmental benefit, as supported by decades of pediatric exercise research (6, 7). International guidelines, including those from the World Health Organization (8) and the U.S. Department of Health and Human Services (9), recommend that children aged 5–17 years accumulate at least 60 min of MVPA daily, incorporating bouts of vigorous aerobic activity on at least 3 days each week. Within this framework, the 2022 Standard’s effort to translate the concept of MVPA into an actionable physiological indicator—the 140–160 bpm range—demonstrates a rare alignment between national education policy and exercise science.

Although no single HR range can capture the diversity of children’s physiological responses, a population-level reference can still serve as a meaningful proxy for MVPA. The intent of such a benchmark is not to prescribe identical workloads for every student but to identify an attainable physiological zone associated with effective exercise intensity across developmental stages. As shown in Table 1, calculations using two applied age-based equations for HR_max_—the widely cited Tanaka et al. formula (10) and the more recent Park et al. (11) model—yield slightly different numerical estimates for school-age children. Yet both indicate that the 140–160 bpm interval corresponds to roughly 65–80 percent of HR_max_ for students aged 7 to 15 years. Although derived from distinct datasets, the two equations converge on the same physiological interpretation: the 2022 Standard’s HR target lies squarely within the MVPA zone recognized internationally for health promotion and youth fitness development (8). In this light, the fixed HR range functions not as an arbitrary numerical rule but as a scientifically defensible and pedagogically practical approximation of effective exercise intensity for the school-age population.

**Table 1:** Estimated proportion of maximal heart rate represented by 140–160 bpm in primary and middle school students based on two predictive equations.

Age (yrs)	Tanaka et al. (10) equation		Park et al. (11) equation			
	Predicted unisex HR_max_ (bpm)	HR zone (%)	Predicted boy’s HR_max_ (bpm)	HR zone for boy (%)	Predicted girl’s HR_max_ (bpm)	HR zone for girl (%)
7	204	(69, 78)	214	(65, 75)	213	(66, 75)
8	203	(69, 79)	213	(66, 75)	212	(66, 75)
9	202	(69, 79)	212	(66, 75)	212	(66, 75)
10	202	(69, 79)	211	(66, 76)	211	(66, 76)
11	201	(70, 80)	210	(67, 76)	210	(67, 76)
12	200	(70, 80)	209	(67, 77)	209	(67, 77)
13	200	(70, 80)	208	(67, 77)	208	(67, 77)
14	199	(70, 80)	207	(68, 77)	208	(67, 77)
15	198	(71, 81)	206	(68, 78)	207	(68, 77)

HR, heart rate; bpm, beats per minute.

The logic behind a fixed HR target also needs to be understood in light of developmental physiology. Children’s cardiovascular systems differ fundamentally from those of adults, exhibiting higher HRs at equivalent workloads due to smaller stroke volumes and reduced cardiac efficiency (12, 13). A 10-year-old, for example, may sustain a HR of 160 bpm at a submaximal oxygen uptake, while an adolescent might reach the same HR at higher relative effort. Such age-dependent variability means that a universal threshold cannot precisely reflect all individual conditions, yet it remains meaningful as an average benchmark that promotes appropriate load for the majority. In this sense, the 2022 Standard is less a rigid prescription than a pragmatic norm—one that seeks to ensure that the average class intensity rises into a scientifically beneficial range without overcomplicating implementation.

Beyond its physiological rationale, the simplicity of the HR-based target also holds pedagogical value. In a classroom setting, easily communicated metrics can foster awareness and engagement. Teachers and students alike benefit from straightforward cues that translate exercise science into daily practice, a principle supported by empirical findings showing that visible feedback mechanisms such as HR monitoring can significantly increase active time and self-regulation among youth (14, 15). The 2022 Standard therefore not only promotes a measurable physiological goal but also encourages an educational culture where effort and health are linked through observable feedback.

Recent pediatric evidence further validates the emphasis on intensity embedded in the 2022 Standard. In a large-scale analysis of youth across the United States, Burden et al. (6) demonstrated that the intensity and duration of daily activity jointly predict cardiorespiratory fitness, with vigorous-intensity bouts contributing disproportionately to aerobic capacity. Similar patterns have been observed in European cohort studies, including HELENA and ALSPAC, where participation in MVPA correlated with superior cardiovascular fitness and metabolic health markers (16, 17). Moreover, experimental and observational studies consistently indicate that intermittent high-intensity activity provides physiological benefits comparable to continuous exercise despite shorter total duration (18, 19). These convergent findings reinforce that reaching and sustaining moderate-to-vigorous intensity, rather than simply extending exercise duration, is central to meaningful physiological adaptation in youth.

The challenge, of course, lies in reconciling scientific precision with the realities of large-scale implementation. Children’s HR responses are influenced not only by biological factors such as maturation and sex but also by contextual elements including emotional excitement, lesson content, and environmental temperature. Two students performing the same task may exhibit markedly different HRs due to fitness levels or engagement. These complexities make a perfectly individualized standard impossible. Nevertheless, as a national policy instrument, the 2022 Standard achieves a delicate balance: it provides direction without excessive complexity and sets a measurable yet attainable target that encourages consistent physiological engagement.

From this view, the 140–160 bpm target should not be dismissed as an oversimplification (5) but appreciated as an intentional policy compromise—one that bridges exercise physiology with educational governance. The 2022 Standard represents one of the first national efforts to embed a biological parameter within curriculum evaluation, elevating PE from a descriptive behavioral activity to an evidence-informed component of public health promotion. This conceptual sophistication underscores China’s determination to root educational reform in scientific reasoning while confronting the realities of diverse school environments. Yet translating such an ambitious vision into classroom practice inevitably exposes new layers of complexity—where differences in resources, interpretation, and local context determine whether a sound physiological idea can truly shape healthier generations.

## 3 Empirical evidence and implementation challenges

While the 140–160 bpm standard rests on sound physiological reasoning, the translation of this benchmark into school practice reveals a more complicated reality. Empirical evidence on the classroom implementation of the 2022 Standard remains limited, yet a recent study using wearable monitors in Chinese primary schools provides valuable preliminary insight. In this investigation, Guo et al. (20) monitored more than 90 PE classes among primary school students in Zhejiang Province, finding that while short bursts of activity reached the prescribed HR range, the overall mean HR across entire lessons typically fell below 140 bpm. Activity type emerged as a decisive factor: fitness and ball games yielded higher intensities, whereas gymnastics and track-and-field sessions often remained in the lower-to-moderate range. This pattern reflects both the pedagogical aims of each activity and the practical challenges teachers face in sustaining continuous exertion within a fixed time frame. Such findings echo international research: studies in Europe and North America report that less than 50 percent of class time typically achieves moderate-to-vigorous intensity even under experienced instruction (21, 22). These converging results suggest that the gap between policy expectation and classroom reality is not uniquely Chinese but rather reflects a global difficulty in sustaining physiologically meaningful intensity within the pedagogical constraints of school PE.

The sources of this gap are multiple and intertwined. First, logistical limitations remain substantial. Most Chinese schools lack HR–monitoring equipment, and where such devices exist, they vary widely in quality, calibration, and maintenance support (23). Even where technology is provided, teachers could face insufficient training in data interpretation, leading to inconsistent use of physiological information during instruction. Without standardized monitoring infrastructure, the HR target risks becoming symbolic rather than functional—an aspirational number instead of a formative feedback tool.

Second, the policy’s interpretive flexibility—initially a strength—can produce confusion in practice. Teachers differ in how they read the “average HR 140–160 bpm” guideline: some interpret it as a momentary target, others as a class-mean requirement, and still others as a personal-effort indicator. These divergent interpretations alter lesson pacing and assessment emphasis. In classes emphasizing technical skill acquisition, HRs naturally fluctuate below the range; in fitness-oriented sessions, HRs may overshoot. This interpretive ambiguity complicates evaluation and may inadvertently pressure teachers to privilege cardiovascular load over pedagogical diversity.

Third, contextual inequities constrain consistent implementation. Regional variation in climate, facility quality, and class size affects achievable intensity. In colder Northern provinces, activity may be reduced for environmental constraints, whereas in warmer or high-altitude regions, HR responses could be elevated due to other environmental stressors. Moreover, large class sizes—often exceeding 40 students—limit individualized monitoring and feedback. These structural disparities illustrate how a scientifically coherent national policy can yield heterogeneous educational outcomes.

Fourth, the technological ecosystem supporting the standard remains underdeveloped. While China has made rapid progress in integrating digital infrastructure into education, few schools adopt wearable devices that estimate HR through optical sensors with variable accuracy. Recent advances in sensor fusion allow simultaneous estimation of HR and oxygen consumption, potentially enabling more precise assessment of load (24). However, widespread adoption of such systems requires investment, teacher training, and standardized data platforms to ensure both validity and privacy. The promise of intelligent monitoring thus coexists with challenges of cost, reliability, and ethical governance.

Despite these barriers, the broader trajectory remains encouraging. The 2022 Standard has catalyzed nationwide experimentation with digital PE management systems, wearable-based lesson evaluation, and data-driven curriculum refinement. Several municipal pilot programs now incorporate automated HR summaries into lesson feedback, allowing teachers to adjust intensity profiles in real time (25). Early reports suggest that classes using feedback-enabled devices achieve significant better physical performance than those using conventional instruction (26, 27). These practical innovations illustrate that the 2022 Standard’s physiological benchmark—while imperfect—has stimulated pedagogical modernization and opened a path toward more objective evaluation.

Ultimately, the experience of implementing the 2022 Standard underscores that educational reform in PE must proceed at the intersection of science, technology, and human practice. The physiological logic of the 140–160 bpm zone remains sound, but its translation requires infrastructure, interpretive clarity, and equitable support. When these elements align, the HR benchmark becomes more than a numeric target: it transforms into a feedback system connecting national policy, classroom behavior, and student health.

## 4 Policy directions

The experience of implementing the 2022 Standard illustrates both the promise and the growing pains of embedding scientific reasoning into national education policy. The inclusion of a physiological indicator in a school curriculum marks a genuine paradigm shift—from defining PE through behavioral participation to grounding it in measurable evidence of exertion. By linking classroom activity to physiological markers, the reform positions PE as an essential component of preventive health and child development. Yet the lessons from early practice (20) reveal that translating scientific standards into equitable outcomes requires not only valid targets but also sustained resources, infrastructure, and policy systems capable of supporting them.

**Figure 1:**
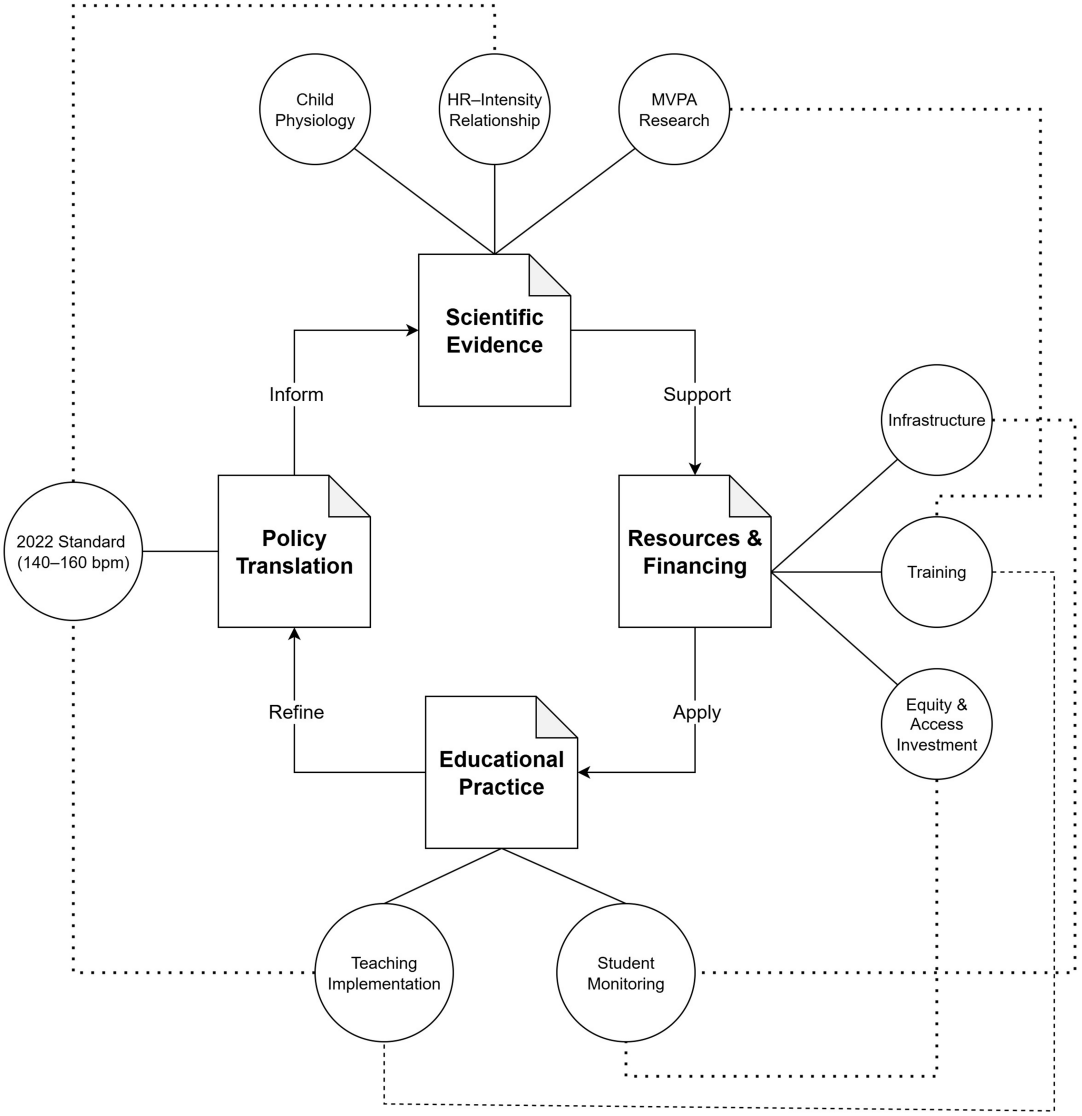
Iterative ecosystem linking science, policy, practice, and resources in the 2022 standard.

Taken together, the emerging evidence indicates that while the 140–160 bpm standard has stimulated meaningful experimentation, its effectiveness depends on the ecosystem surrounding implementation. Effective implementation depends on four interconnected dimensions: scientific precision, pedagogical adaptation, technological integration, and sustainable resourcing. Each must evolve in concert for the policy to reach its full potential. Figure 1 conceptually illustrates this emerging, interdependent ecosystem, from policy formulation to real-world application.

First, scientific precision should be refined through ongoing monitoring and age-specific calibration. Current evidence shows that inter-individual differences in sex-related growth and fitness can shift HR responses by as much as 10 bpm at a given workload (21). Establishing reference distributions by age, sex, and region (e.g., high altitude provinces) would allow the national benchmark to evolve from a single fixed range to an adaptive system with recommended sub-zones. Collaboration among sports scientists, pediatricians, and educators could generate a living database that updates the HR target as more wearable-based data accumulate across provinces. In this sense, the 2022 Standard can become not a static prescription but a continuously improving evidence platform.

Second, pedagogical adaptation is essential for transforming physiological guidance into meaningful learning. Teachers remain the pivotal agents linking data with action. When HR feedback is used merely as a numeric compliance check, it risks narrowing pedagogy to pursuit of the number. However, when interpreted as a teaching tool—informing pacing, rest intervals, or skill progressions—it enriches both instruction and engagement. Incorporating HR interpretation and basic physiology into teacher-training curricula would thus reinforce the 2022 Standard’s educational intent: promoting health literacy rather than mechanical measurement.

Third, technological integration remains the linchpin for large-scale feasibility. The rapid development of low-cost optical HR sensors and hybrid algorithms that estimate health status provides an unprecedented opportunity for objective monitoring (28). However, technology alone cannot solve the human and ethical challenges of data use. Schools need secure data platforms that protect privacy, ensure transparency of algorithmic calculations, and prevent competitive misuse of physiological information. National-level guidelines on data storage, consent, and reporting could safeguard trust while enabling meaningful research collaboration. A transparent digital ecosystem would allow educators, policymakers, and scientists to share validated metrics without compromising student rights.

Fourth, resources and financing form the indispensable foundation beneath all these efforts. The cost of monitoring devices, data infrastructure, and teacher training continues to limit nationwide consistency. Ensuring fiscal support for equipment procurement, technical maintenance, and teacher incentives is essential if physiological monitoring is to become a sustainable component of education rather than a short-term experiment. In this regard, equitable resource allocation represents not only an economic issue but a matter of health justice for all students.

Looking ahead, three priorities appear most urgent. First, national and provincial agencies should invest in longitudinal monitoring programs linking HR-derived indicators to tangible health outcomes such as aerobic fitness, body composition, and academic wellbeing. Evidence of causal benefit will strengthen public confidence in physiologically guided education. Second, interdisciplinary collaboration should be institutionalized through joint working groups between the ministries of education, health, and technology to coordinate standards for devices, data, teacher training, and resource allocation. Third, greater attention should be given to equity: rural and resource-limited schools must receive targeted technical and financial support, reinforcing the resources and financing foundation emphasized in this perspective, to prevent the widening of health disparities through differential access to monitoring tools.

Although this review provides a broad and integrative perspective, several limitations should be acknowledged. The analysis relies on published literature and early implementation reports rather than longitudinal empirical data, restricting causal inference and long-term evaluation of outcomes. Additionally, as wearable-technology access and data processing differ across schools, physiological monitoring data should be interpreted cautiously until national validation systems and consistent quality assurance frameworks are established.

## 5 Global implications

Beyond China, the reform offers a valuable model for other nations seeking to modernize PE curricula. Many systems still evaluate success through attendance or subjective effort ratings. A scientifically anchored yet flexible metric—calibrated to local developmental and cultural contexts—could improve both accountability and health outcomes. The Chinese experience demonstrates that even a single numeric standard can catalyze large-scale reflection on what constitutes effective activity in childhood. As more countries adopt wearable technologies and digital curricula, the challenge will shift from measurement toward meaning: how to interpret physiological data in ways that enrich pedagogy rather than reduce it to surveillance. Although several nations have emphasized the promotion of MVPA in schools, few have institutionalized a physiological benchmark comparable to China’s. The United States and many European countries adopt curriculum guidelines encouraging MVPA for at least 50% of class time, but enforcement typically depends on observational evaluation rather than objective measurement. The United Kingdom’s “Daily Mile” initiative (29) and the European Union’s MOVING framework similarly encourage activity monitoring without specifying heart-rate zones. These systems highlight two recurring challenges: the absence of standardized physiological indicators and the logistical burden of equipping schools with reliable monitoring technology. Implementing similar policies elsewhere would require incremental approaches—beginning with pilot programs that validate feasible HR targets, teacher-training in physiological monitoring, and development of national data platforms that ensure privacy and equity. Such progressive adoption could enable other countries to translate scientific evidence into actionable, context-sensitive PE policies.

## 6 Conclusion

In the long term, the true legacy of the 2022 Standard may lie not in its specific HR range but in its underlying philosophy—that child health is measurable, improvable, and inseparable from the quality of daily schooling. The 140–160 bpm benchmark, though imperfect, symbolizes a new era in which educational excellence and physiological understanding converge. Its success will depend on maintaining this balance: preserving the scientific rigor that inspired it while embracing the pedagogical, technological, and financial flexibility that ensures its sustainability.
